# Research on asphalt pavement structure with cement treated large size macadam (CTB–50) base course

**DOI:** 10.1371/journal.pone.0325210

**Published:** 2025-07-07

**Authors:** Bentao Zhou, Yuan Yao, Xiaoke Geng, Yingjun Jiang, Tiezheng Guan, Ji Liang, Yue Wang

**Affiliations:** 1 Henan Jiaotou Shangluo Expressway Co., Ltd., Zhengzhou, Henan, China; 2 Henan Transport Investment Group Co., Ltd., Zhengzhou, Henan, China; 3 Key Laboratory for Special Area Highway Engineering of Ministry of Education, Chang’an University, Xi’an, Shaanxi, China; 4 Henan Zhongtian High-tech Smart Technology Co., Ltd., Zhengzhou, Henan, China; 5 China Railway No.5 Engineerging Group Co., Ltd., Changsha, Hunan, China; Shandong University of Technology, CHINA

## Abstract

Cement-treated large-size macadam base (CTB–50) has a high modulus, high strength, and good durability, which can increase the paving and rolling thickness, reduce the base layers, and enhance the overall structure of the pavement structure. However, there are no studies conducted on the mechanical response of asphalt pavement with a CTB–50 base course such that it can be promoted and applied in pavement engineering using CTB–50. This study analyzed the mechanical response and fatigue life of asphalt pavement structures with different base courses. Subsequently, the typical pavement structure form of asphalt pavement with a CTB–50 base course was recommended, and its effectiveness was verified through two highway engineering projects. The results showed that the base structure reduced from a three-layer CTB–30 base course structure to a two-layer CTB–50 base course structure, and the base layer bottom tensile stress of the asphalt pavement with CTB–50 base course is reduced by 5% compared to that of the asphalt pavement with CTB–30 base course. Based on the principle of equivalent fatigue life, the CTB–50 base layer thickness of the asphalt pavement can be reduced by approximately 6 cm compared to that of the CTB–30 base layer. The recommended pavement structure can reduce one layer in the construction of the base layer and improve the integrity of the asphalt pavement structure. The experimental road paved with the recommended CTB–50 base course pavement structure showed no evident pavement disease, whereas the road section with the CTB–30 base course showed early crack diseases in the asphalt pavement.

## 1. Introduction

Semi-rigid base asphalt pavement is the most common pavement structure type in China [1], and approximately 75% of its base materials are cement-treated base materials with a nominal maximum aggregate size of 31.5 mm (CTB–30) [2]. However, the major distress of semi-rigid base asphalt pavement is the cracking of semi-rigid base, which causes propagation of cracks in the asphalt pavement structure; therefore, resolving the distress of cracking in semi-rigid bases has become a research hotspot [3,4]. The anti-cracking of semi-rigid base materials are attributed to several factors, such as cement content, maximum aggregate size, gradation, water content, and degree of compaction [5]. Extensive research has been conducted to improve the crack resistance of semi-rigid base materials.

Priastiwi et al. [6] studied the effect of cement content on the compressive strength of a cement-treated base and showed that although increase in cement content can improve the compressive strength of the cement-treated base, it should be minimized as much as possible to make the cement-treated base more economical and resistant to cracking. Increasing the nominal maximum aggregate size and the content of coarse aggregate in the mixture and adopting a strong interlocking skeleton-dense gradation of cement-treated macadam base material can enhance the core aggregate embedded extrusion force and mechanical strength of the mixture, which can reduce the cement content and improve the anti-cracking of semi-rigid base materials [7,8]. Ding proposed that the base course of an asphalt pavement transformed from three layers to two layers can significantly improve the interlayer bonding state and reduce the base layer bottom tensile stress [9,10].

Previous studies have shown that using cement-treated large-sized macadam base material, the coarse aggregate-embedded extrusion can be improved and the cement content can be reduced; thus, the cement-treated macadam base material has stronger mechanical strength, better anti-cracking performance, and volume stability. Jiang et al. [11,12] studied the compressive and splitting strengths of cement-treated base materials with a nominal maximum aggregate size of 53 mm (CTB–50). Results demonstrated that the mechanical properties of CTB–50 were improved by at least 10% than that of the traditional cement-treated base material CTB–30. The cement content of CTB–50 was less than that of CTB–30 under the same mechanical strength control index, thereby achieving the goal of better anti-cracking properties and lower engineering costs. This provides theoretical support for the promotion and application of CTB–50 in pavement engineering.

However, increasing the nominal maximum aggregate size and coarse aggregate content of cement-treated base materials can lead to mixture segregation during the construction of the base course [13], which imposes higher requirements for construction control. The level of construction machinery is developing, and the anti-segregation ability of construction machinery has been significantly improved [14,15], which also supports the application of CTB–50 in pavement engineering.

Therefore, to verify whether CTB–50 can replace traditional CTB–30 and be applied to semi-rigid base asphalt pavement structures, a stress characteristic analysis of asphalt pavement structures with a CTB–50 base course is necessary. Assogba et al. [16] investigated the distribution of mechanical parameters in different semi-rigid pavement structures with a CTB–30 base course or sub-base course based on 3D pavement FE modeling. The field mechanical response and calculated mechanical response of the finite element method (FEM) with different CTB–30 base-course modulus inputs were evaluated by Qian et al. [17].

Most existing studies have only considered the mechanical response of asphalt pavement with a CTB–30 base course or a subbase course. However, no studies have been conducted on the mechanical response of asphalt pavement with a CTB–50 base course, which is unfavorable for the promotion and application of CTB-50 in pavement engineering. The present study aims to analyze the mechanical response of asphalt pavement with a CTB–50 base course and a CTB–30 base course. The typical pavement structure form of asphalt pavement with a CTB–50 base course was recommended, and its effectiveness was verified through two highway engineering projects. This study effectively mitigates base course cracking while simultaneously lowering project costs. Based on our findings, this study recommends the CTB-50 base course structure for asphalt pavement applications, thereby providing theoretical support for its broader engineering implementation.

## 2. Test program

**Fig 1:**
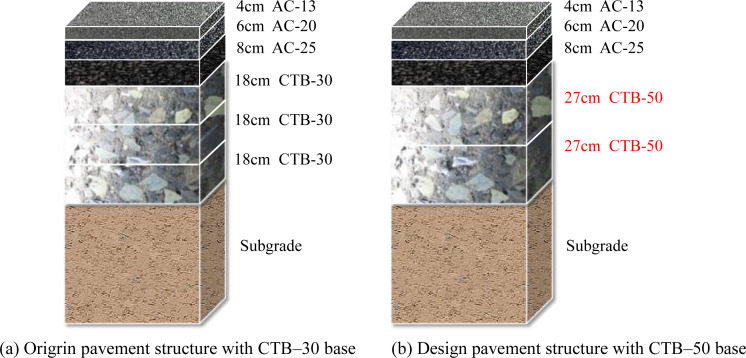
Pavement structure of Yao Luan Xi Expressway highway. (a) Origrin pavement structure with CTB–30 base (b) Design pavement structure with CTB–50 base.

**Fig 2:**
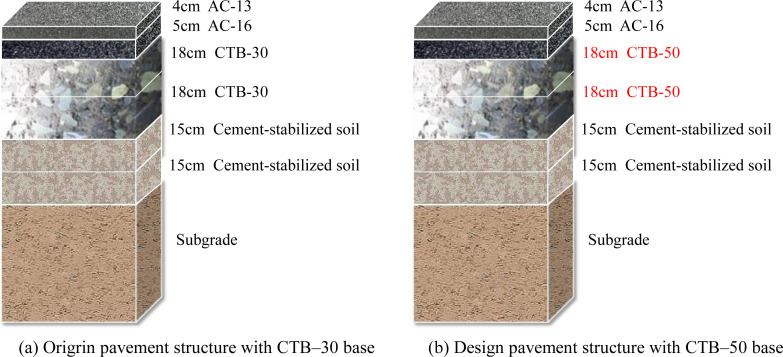
Pavement structure of G234 second–class highway. (a) Origrin pavement structure with CTB–30 base (b) Design pavement structure with CTB–50 base.

In China, highways can be divided into high- and low-grade highways, of which expressways and first-class highways belong to high-grade highways, and second- and lower-class highways belong to low-grade highways [18]. Two different classes of highway projects were selected to analyze the mechanical response and fatigue life of asphalt pavement with CTB–50 and CTB–30 base courses. The Yao Luan Xi Expressway highway project, which represents the expressway and first-class highway, was built according to the twin four-lane expressway highway and 80 km/h design speed; its design pavement structure is shown in Fig 1. The G234 second-class highway reconstruction project, which represents the second- and lower-class highways, was built according to the twin two-lane highway and a 60 km/h design speed; its pavement structure is shown in Fig 2.

As shown in Figs 1 and 2, the pavement structure of an expressway consists of three layers of surface courses and three layers of base courses, whereas the pavement structure of a second-class highway consists of two layers of surface courses and two layers of base courses. The base layer of the original pavement structure was CTB–30. To study the influence of the CTB–50 base layer on the performance of the asphalt pavement, CTB–50 is adopted instead of CTB–30 of the original pavement structure, and the asphalt pavement structure with a CTB–50 base course is recommended in Fig 2. The nominal maximum particle size of the base course material was expanded from 31.5 to 53 mm and a two-layer CTB–50 base course was added to the asphalt pavement structure.

### 2.1 Calculation model and parameters

**Fig 3:**
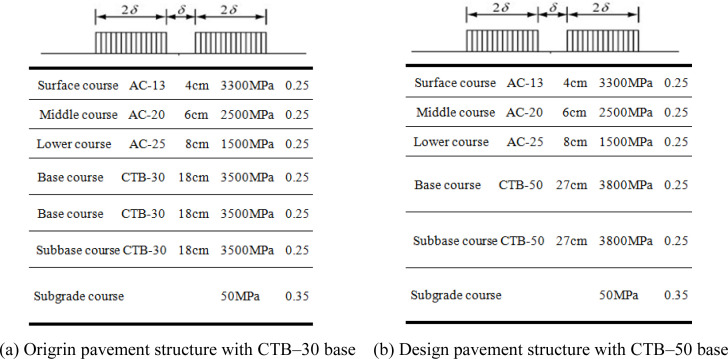
Mechanical calculation model of Expressway highway. (a) Origrin pavement structure with CTB–30 base (b) Design pavement structure with CTB–50 base.

**Fig 4:**
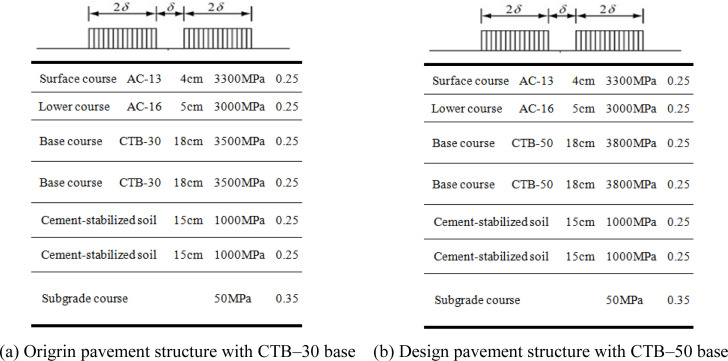
Mechanical calculation model of second–class highway. (a) Origrin pavement structure with CTB–30 base (b) Design pavement structure with CTB–50 base.

BISAR software (version 3.0) was used to analyze the mechanical responses of the different asphalt pavement structures. The calculated load was the standard axle load BZZ–100 with a double circular uniform vertical distribution [19,20]. The mechanical calculation model and parameters of the pavement structure are shown in Figs 3 and 4.

The asphalt surface layer was assumed to be in a fully continuous contact state, whereas the cement-stabilized crushed stone and cement-stabilized soil layers were assumed to be in a semi-continuous contact state.

### 2.2 Selection of interlayer contact state parameters

AK was used to represent the interlayer contact state in BISAR software version 3.0, and the AK values for the fully continuous, semi-continuous, and completely smooth states were 0, 0.5 × 10^−9^, and 1 × 10^−9^ m^3^/N, respectively.

### 2.3 Selection of calculation point

**Fig 5:**
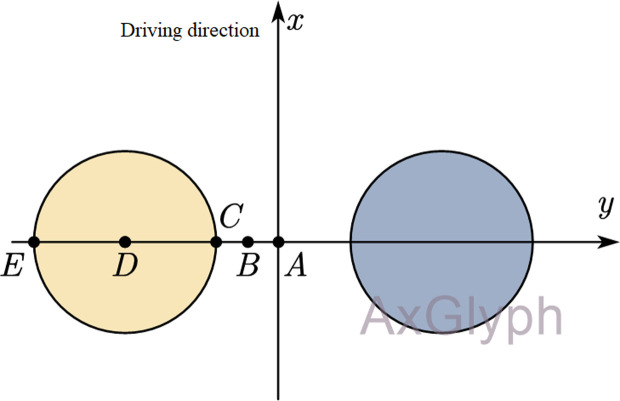
Mechanical responses calculation points.

Assuming that the x-axis is the loading driving direction, the center distance between the two wheels is fixed at 319.5 mm, and the load circle radius is 106.5 mm, the five selected mechanical response calculation points and their coordinates are points A (0,0), B (−0.0266,0), C (−0.0533,0), D (−0.1598,0), and E (−0.2663,0), as shown in Fig 5.

**Fig 6:**
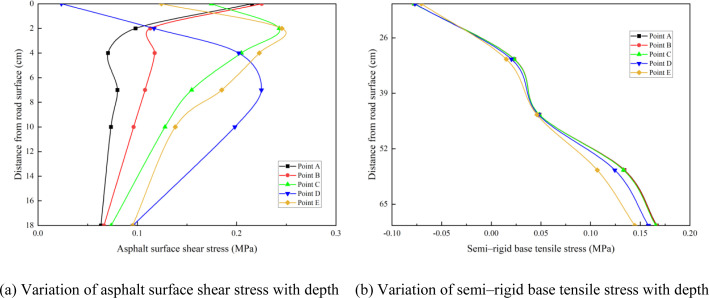
Variation of mechanical responses with depth of different calculation points. (a) Variation of asphalt surface shear stress with depth (b) Variation of semi–rigid base tensile stress with depth.

The calculation results for the expressway highway asphalt surface shear stress and semi-rigid base tensile stress at different depths based on the above-mentioned model are shown in Table 1, and the variations in the asphalt surface shear stress and semi-rigid base tensile stress with depth are shown in Fig 6.

**Table 1:** Mechanical responses calculation results of different points.

Items	Distance from road surface (cm)	Stress of different points (MPa)				
		A	B	C	D	E
**Asphalt surface shear stress**	0	0.2155	0.2253	0.1746	0.0236	0.1242
	2	0.0982	0.1131	0.2428	0.1168	0.2455
	4	0.0705	0.1175	0.2047	0.2020	0.2228
	7	0.0800	0.1078	0.1547	0.2249	0.1850
	10	0.0735	0.0963	0.1279	0.1982	0.1382
	18 (Surface layer bottom)	0.0634	0.0665	0.0737	0.0956	0.0953
**Semi–rigid base tensile stress**	18	–0.0775	–0.0776	–0.0776	–0.0769	–0.0703
	31	0.0232	0.0231	0.0229	0.0203	0.0151
	45 (base layer bottom)	0.1341	0.1338	0.1331	0.1244	0.1067
	58	0.0485	0.0485	0.0484	0.0475	0.0460
	72 (Subbase layer bottom)	0.1666	0.1663	0.1656	0.1582	0.1444

Fig 6(a) shows that locations with high shear stress in the asphalt surface layer occur at points E and D. Point E is approximately 2–4 cm from the road surface and point D is approximately 6–8 cm from the road surface. As shown in Fig 6(b), the maximum tensile stress of the semi-rigid base appeared at point A at the bottom of the base. Therefore, points D and E were selected as the shear stress calculation points for the asphalt surface layer, and point A was selected as the tensile stress calculation point for the semi-rigid base layer.

### 2.4 Selection of evaluation parameter

Previous studies have shown that the shear stress of asphalt pavements is related to rutting, and the base layer bottom tensile stress is related to the fatigue cracking of the pavement [21]. Therefore, the influence of the thickness and modulus of the cement-stabilized crushed stone on the maximum shear stress of the asphalt pavement and the maximum tensile stress of the semi-rigid base was studied. The results are presented in Tables 2 and 3.

**Table 2:** Mechanical responses of different base thickness.

Base thickness (cm)	50	52	54	56	58
**Base modulus (MPa)**	3800				
**τ**_**max**_ **(MPa)**	0.2463	0.2465	0.2468	0.2472	0.2477
**σ**_**t**_ **(MPa)**	0.1865	0.1707	0.1585	0.1488	0.1392

**Table 3:** Mechanical responses of different base modulus.

Base thickness (cm)	54				
**Base modulus (MPa)**	3000	3200	3400	3600	3800
**τ**_**max**_ **(MPa)**	0.2480	0.2477	0.2474	0.2471	0.2468
**σ**_**t**_ **(MPa)**	0.1422	0.1465	0.1506	0.1546	0.1585

From Tables 2 and 3, it can be observed that as the thickness and modulus of the base layer increased, the maximum shear stress of the asphalt surface layer remained unchanged. However, the maximum tensile stress of the semi-rigid base layer significantly decreased with the thickness of the base layer increased, and the maximum tensile stress of the semi-rigid base layer significantly increased with the modulus of the base layer increased. This indicates that the tensile stress of the semi-rigid base layer is sensitive to changes in the thickness and modulus of the base layer. Therefore, the base layer bottom tensile stress of the semi-rigid base layer was selected as the mechanical response evaluation index in this study.

## 3. Mechanical response analysis of asphalt pavement with CTB–50 base course

### 3.1 Influence of base modulus on layer bottom tensile stress of pavement base layer

The base layer bottom tensile stresses under different asphalt pavement structures are listed in Table 4.

**Table 4:** Base layer bottom tensile stress under different asphalt pavement structure.

Pavement structure type	Base modulus (MPa)	Base layer bottom tensile stress of different pavement structure (MPa)	
		G234 second–class highway	Yaoluanxi Expressway highway
CTB–30	3500	0.1933	0.1670
CTB–50	3800	0.2056	0.1585

It can be noted from Table 4 that for second-class highway, when the pavement structure of surface layer, the thickness of the base layer, and the number of construction layers are the same, the base layer bottom tensile stress increases with an increase in the base material modulus. The base layer bottom tensile stress of the asphalt pavement with CTB–50 base course increases by 6% compared with that of the asphalt pavement with CTB–30 base course; whereas for expressway highways, the base layer bottom tensile stress of the asphalt pavement with CTB–50 base course was reduced by 5% compared with that of the asphalt pavement with CTB–30 base course. This was because the expressway highway base structure reduced from three-layer CTB–30 base structure to two-layer CTB–50 base structure, which enhanced the overall integrity of the pavement structure [22]. Although an increase in the base material modulus increases the base layer bottom tensile stress, the effect of reducing the base layer bottom tensile stress through the enhancement of the structural integrity is greater than that of increasing the modulus, resulting in a decrease in the base layer bottom tensile stress of expressway highway asphalt pavements.

### 3.2 Influence of base thickness on layer bottom tensile stress of expressway and first-class highway

The base layer bottom tensile stresses of the expressway and first-class highway at different base thicknesses are listed in Table 5. Considering that the base thickness of the semi-rigid base of the expressway and first-class highway is 46–56 cm, the CTB–30 base adopts a three-layer structure, whereas the CTB–50 base adopts a two-layer structure.

**Table 5:** Base layer bottom tensile stress under different base thickness of express highway.

Pavement structure type	Base layer bottom tensile stress (MPa)of different base thickness (cm)				
	46	48	51	54	56
**CTB–30**	0.2065	0.1924	0.1792	0.1670	0.1603
**CTB–50**	0.1982	0.1844	0.1708	0.1585	0.1512

**Fig 7:**
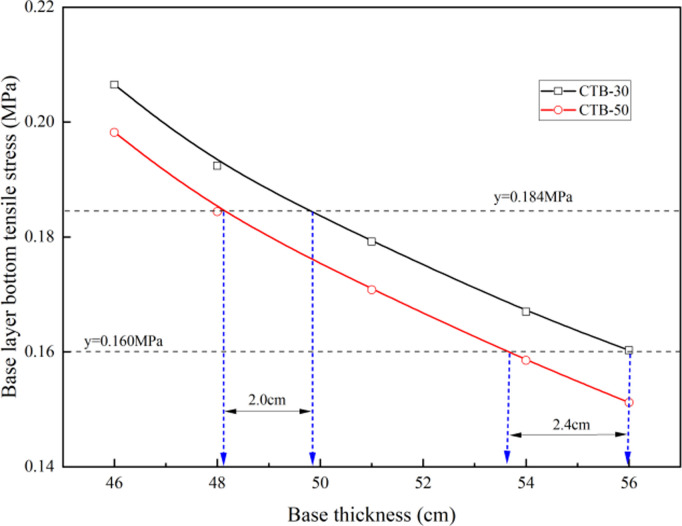
Variation of base layer bottom tensile stress with different base thickness of express highway.

As shown in Fig 7, for asphalt pavement with CTB–50 base course or CTB–30 base course, the base layer bottom tensile stress under load gradually decreases with the increase in base thickness. If the base layer bottom tensile stress is the same, the CTB–50 base layer thickness of the asphalt pavement can be reduced by approximately 2 cm compared to that of the CTB–30 base layer; as the base layer thickness of the asphalt pavement increases, the CTB–50 base layer thickness of the asphalt pavement can also be reduced more.

### 3.3 Influence of base thickness on layer bottom tensile stress of second-class highway

The base layer bottom tensile stresses of the second-class highways with different base thicknesses are listed in Table 6. Considering that the base thickness of semi-rigid base of the second-class highway is 30–40 cm, both the CTB–30 and CTB–50 bases adopt a two-layer structure.

**Table 6:** Base layer bottom tensile stress under different base thickness of second–class highway.

Pavement structure type	Base layer bottom tensile stress (MPa)of different base thickness (cm)					
	30	32	34	36	38	40
**CTB–30**	0.2244	0.2137	0.2033	0.1933	0.1837	0.1746
**CTB–50**	0.2392	0.2276	0.2164	0.2056	0.1952	0.1854

**Fig 8:**
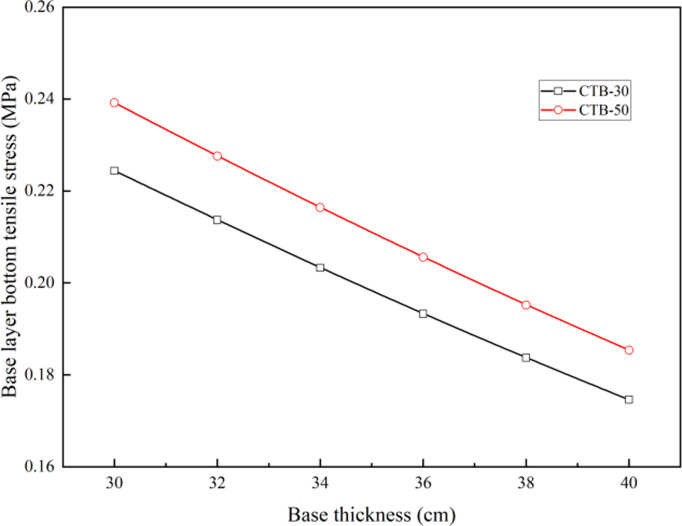
Variation of base layer bottom tensile stress with different base thickness of second–class highway.

The correlation analysis between base layer bottom tensile stress and base thickness is shown in Fig 8, demonstrating that when the number of base layers of the asphalt pavement is same, the base layer bottom tensile stress of the asphalt pavement with CTB–50 base course increases by approximately 6% compared to that of the asphalt pavement with CTB–30 base course, owing to the high modulus of CTB–50.

From the above results, it can be observed that if the thickness of the single base layer increases, the base course of asphalt pavement can be transformed from three-layer CTB–30 base course into two-layer CTB–50 base course, which enhances the overall integrity of the asphalt pavement base and reduces the base layer bottom tensile stress; however, if the thickness of the single base layer remains unchanged, the advantage of the CTB–50 base layer with large-thickness paving cannot be demonstrated, and the base layer bottom tensile stress increases.

Previous research has shown that the design of asphalt pavement structures should not only analyze the load stress of the structural layer but also consider the strength of the pavement materials. Moreover, the fatigue characteristics of materials depend on the material strength and load stress, which are stress levels. Therefore, analyzing the load stress of the structural layer cannot accurately determine whether the base layer experiences fatigue cracking. Therefore, this study comprehensively analyzes the load stress and material strength of the base layer and estimates the fatigue life of the CTB–50 base layer based on the fatigue equation.

## 4. Fatigue life analysis of asphalt pavement with CTB–50 base course

In conventional practice, the cement content ranges from 2.5% to 4.0% for CTB–30 and 2.0% to 3.0% for CTB–50. For engineering applications, a cement content of 2.5% is commonly adopted in CTB–50. To ensure a consistent and meaningful comparison of the fatigue performance between CTB–50 and CTB–30, the fatigue curves of CTB–30 and CTB–50 under 50% failure probability and 2.5% cement content are adopted in this study.


CTB−30:lgN=1.457−20.964lgS (1)

CTB−50:lgN=1.572−21.097lgS (2)

where N is fatigue life of the material and S is stress level.

It can be observed from Eqs. (1) and (2), the intercept of the fatigue equation curve on the ordinate axis of CTB–50 is higher than that of CTB–30, which indicates the better fatigue performance of CTB–50 [23,24].

### 4.1 Stress level analysis of asphalt pavement with CTB–50 and CTB–30 base courses

#### 4.1.1 Stress level analysis of expressways and first-class highway asphalt pavements.

**Fig 9:**
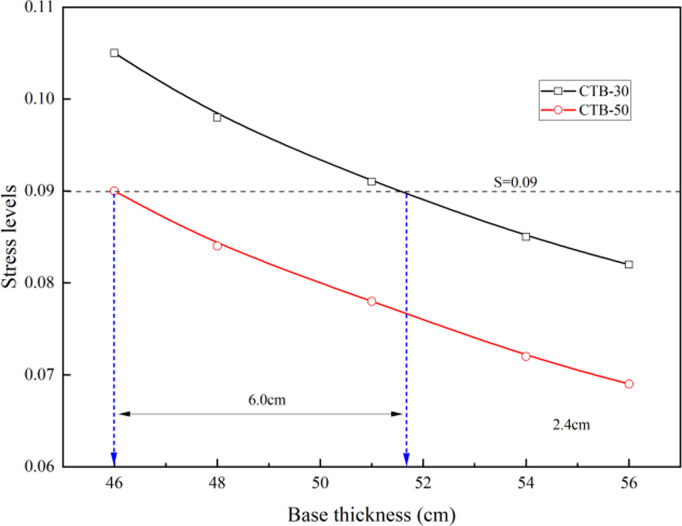
Variation of stress levels with different base thickness of express highway.

The stress levels of the expressway and first-class highway asphalt pavement with CTB–30 and CTB–50 base layers are presented in Table 7 and Fig 9. The splitting strengths of CTB–30 and CTB–50 are 1.40 and 1.57 MPa, respectively, and the flexural strength is 1.4 times the splitting strength.

**Table 7:** Stress level of expressway and first–class highway asphalt pavement.

Pavement structure type	Items	Mechanical indicators of different base thicknesses (cm)				
		46	48	51	54	56
**CTB–30**	**Splitting strengths (MPa)**	1.40				
	**Flexural strength (MPa)**	1.96				
	**Base layer bottom tensile stress (MPa)**	0.2065	0.1924	0.1792	0.1670	0.1603
	**Stress levels**	0.105	0.098	0.091	0.085	0.082
**CTB–50**	**Splitting strengths (MPa)**	1.57				
	**Flexural strength (MPa)**	2.20				
	**Base layer bottom tensile stress (MPa)**	0.1982	0.1844	0.1708	0.1585	0.1512
	**Stress levels**	0.090	0.084	0.078	0.072	0.069

The stress levels of the expressway and first-class highway asphalt pavement with CTB–50 base course decreased by approximately 15% compared to the asphalt pavement with CTB–30 base course; and if the stress level is the same, the CTB–50 base layer thickness of asphalt pavement can be reduced by approximately 6 cm compared to that of CTB–30 base layer.

#### 4.1.2 Stress level analysis of second-class highway asphalt pavement.

**Fig 10:**
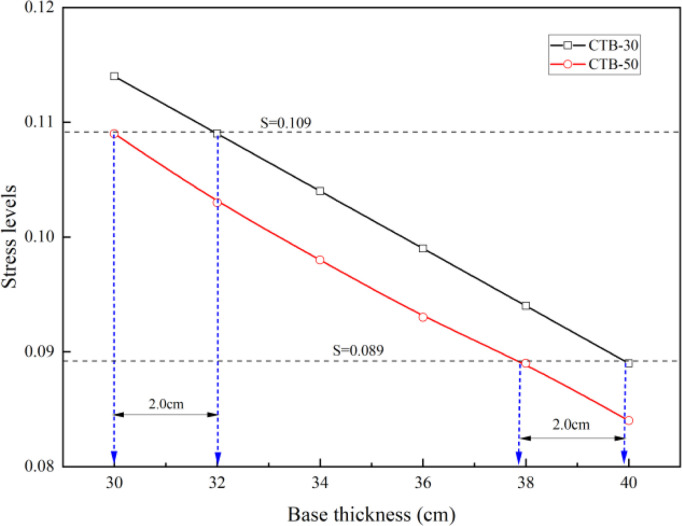
Variation of stress levels with different base thickness of second–class highway.

The stress levels of the second-class highway asphalt pavements with CTB–30 and CTB–50 base layers are presented in Table 8 and Fig 10.

**Table 8:** Stress level of second–class highway asphalt pavement.

Pavement structure type	Items	Mechanical indicators of different base thicknesses (cm)					
		30	32	34	36	38	40
**CTB–30**	**Splitting strengths (MPa)**	1.40					
	**Flexural strength (MPa)**	1.96					
	**Base layer bottom tensile stress (MPa)**	0.2244	0.2137	0.2033	0.1933	0.1837	0.1746
	**Stress levels**	0.114	0.109	0.104	0.099	0.094	0.089
**CTB–50**	**Splitting strengths (MPa)**	1.57					
	**Flexural strength (MPa)**	2.20					
	**Base layer bottom tensile stress (MPa)**	0.2392	0.2276	0.2164	0.2056	0.1952	0.1854
	**Stress levels**	0.109	0.103	0.098	0.093	0.089	0.084

As shown in Table 8, although the base layer bottom tensile stress of the second-class highway asphalt pavement with CTB–50 base course was greater than that of the asphalt pavement with CTB–30 base course, owing to the high strength of the CTB–50 material, the stress levels of the second-class highway asphalt pavement with CTB–50 base course decreased by approximately 6% compared to the asphalt pavement with CTB–30 base course. If the stress level is the same, the CTB–50 base layer thickness of the second-class highway asphalt pavement can be reduced by approximately 2 cm compared with that of the CTB–30 base layer.

### 4.2 Fatigue life of asphalt pavement with CTB–50 and CTB–30 base courses

#### 4.2.1 Fatigue life of expressway and first-class highway asphalt pavements.

**Fig 11:**
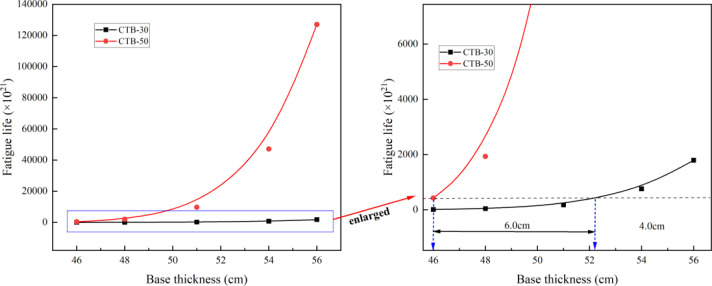
Variation of fatigue life with different base thickness of express highway.

The fatigue life of the expressway and first-class highway asphalt pavements with CTB–30 and CTB–50 base layers are presented in Table 9 and Fig 11.

**Table 9:** Fatigue life of expressway and first–class highway asphalt pavement.

Pavement structure type	Fatigue life (×10^21^) of asphalt pavement with different base thicknesses (cm)				
	46	48	51	54	56
**CTB–30**	8.8	39	173	757	1790
**CTB–50**	422	1930	9730	47100	127000

As shown in Fig 11, the thicker the base layer, the higher the fatigue life of the cement-stabilized crushed stone base layer, and the fatigue life of the CTB–50 base layer is significantly greater than that of CTB–30. If the fatigue life of the CTB–50 base layer is equal to that of CTB–30, the CTB–50 base layer thickness of expressway and first-class highway asphalt pavements can be reduced by approximately 2 cm compared to that of the CTB–30 base layer.

The thickness of semi-rigid base of the expressway and first-class highway was 46–56 cm. Based on the principle of equivalent fatigue life, the CTB–50 base layer thickness of the expressway and first-class highway asphalt pavement can be reduced to 40–50 cm. The base course can be constructed in two layers; thus, the base course of the asphalt pavement can be transformed from a three-layer CTB–30 base course into a two-layer CTB–50 base course, which eliminates the construction of one layer of the base layer.

#### 4.2.2 Fatigue life of second-class highway asphalt pavement.

**Fig 12:**
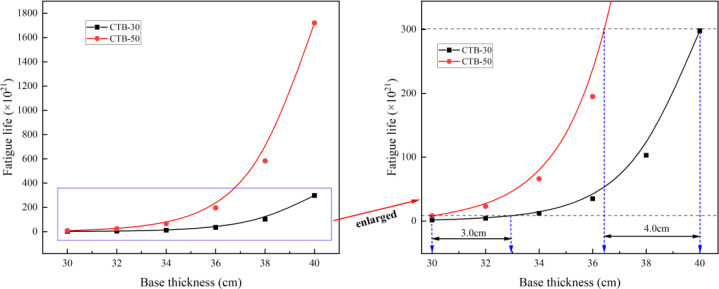
Variation of fatigue life with different base thickness of second highway.

The fatigue life of the second-class highway asphalt pavements with CTB–30 and CTB–50 base layers is presented in Table 10 and Fig 12.

**Table 10:** Fatigue life of second–class highway asphalt pavement.

Pavement structure type	Fatigue life (×10^21^) of asphalt pavement with different base thicknesses (cm)					
	30	32	34	36	38	40
**CTB–30**	1.6	4.3	12	35	103	298
**CTB–50**	8	23	66	195	582	1720

Based on the principle of equivalent fatigue life, the CTB–50 base layer thickness of second-class highway asphalt pavement can be reduced by approximately 3–4 cm compared to that of the CTB–30 base layer.

## 5. Research on asphalt pavement structure with CTB–50 base course

### 5.1 Expressway and first-class highway asphalt pavement structure with CTB–50 base course

**Fig 13:**
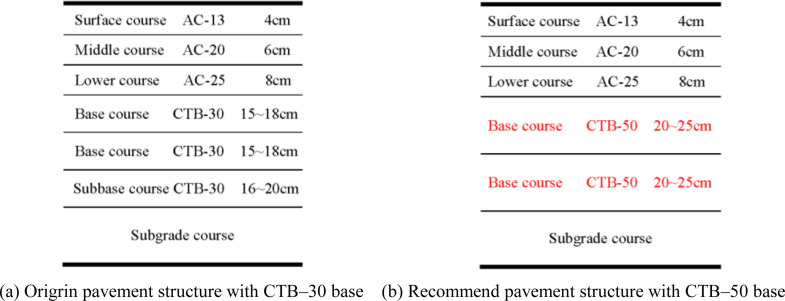
Recommended pavement structure of expressway highway. (a) Origrin pavement structure with CTB–30 base (b) Recommend pavement structure with CTB–50 base.

The thickness of semi-rigid base of the expressway and first-class highway was 46–56 cm. Based on the principle of equivalent fatigue life, the CTB–50 base layer thickness of expressway and first-class highway asphalt pavements can be reduced by approximately 6 cm compared with that of the CTB–30 base layer. An expressway and first-class highway asphalt pavement structure with a CTB–50 base course was recommended, as shown in Fig 13.

It can be noted from Fig 13 that the asphalt pavement structure of the expressway and first-class highway with the CTB–50 base course maintains the asphalt pavement layers unchanged; the base course of the asphalt pavement was transformed from three-layer CTB–30 base course into two-layer CTB–50 base course, and the CTB–50 base layer thickness of the expressway and first-class highway asphalt pavement was reduced to 40–50 cm. This eliminated the construction of one layer of the base layer and improved the integrity of the asphalt pavement structure.

### 5.2 Second-class highway asphalt pavement structure with CTB–50 base course

**Fig 14:**
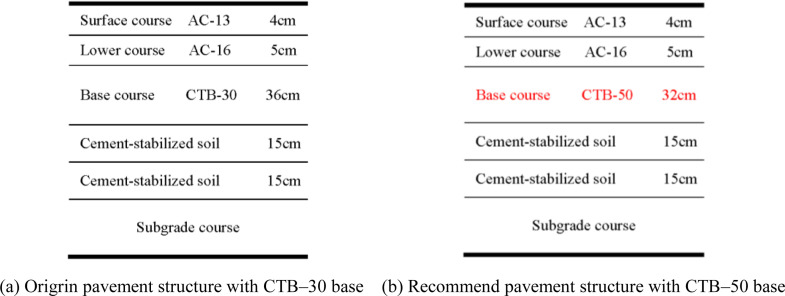
Pavement structure based on the principle of equivalent fatigue life of second–class highway. (a) Origrin pavement structure with CTB–30 base (b) Recommend pavement structure with CTB–50 base.

The thickness of the semi-rigid base of the second-class highway was 46–56 cm. Based on the principle of equivalent fatigue life, the CTB–50 base layer thickness of the second-class highway asphalt pavement can be reduced by approximately 4 cm compared with that of the CTB–30 base layer. Subsequently, a second-class highway asphalt pavement structure with a CTB–50 base course was recommended, as shown in Fig 14.

As shown in Fig 14, the second-class highway asphalt pavement structure with the CTB–50 base course maintained the asphalt pavement layers unchanged; the CTB–50 base layer thickness of the second-class highway asphalt pavement structure was reduced to 32 cm.

However, considering that the maximum rolling thickness of the compaction equipment during the construction of the base course was 28 cm, if the CTB–50 base thickness was 32 cm, the base course would still need to be constructed in two layers. From previous research, it can be noted that if the number of base courses is the same, although the fatigue life of CTB–50 base layer of thickness 32 cm is equal to that of the CTB–30 base layer of thickness 36 cm, the base layer bottom tensile stress of the asphalt pavement with the CTB–50 base layer will be greater than that of the CTB–30 base layer owing to the increase in the base material modulus. This is because the two-layer paving does not fully utilize the advantages of CTB–50 in large-thickness paving and rolling; the pavement overall structure is not enhanced, and the rolling thickness is relatively small; therefore, it can easily cause aggregate crushing during the compaction process. Therefore, the adoption of a CTB–50 base course of thickness 32 cm for the second-class highway asphalt pavements is not the best asphalt pavement structure scheme.

**Fig 15:**
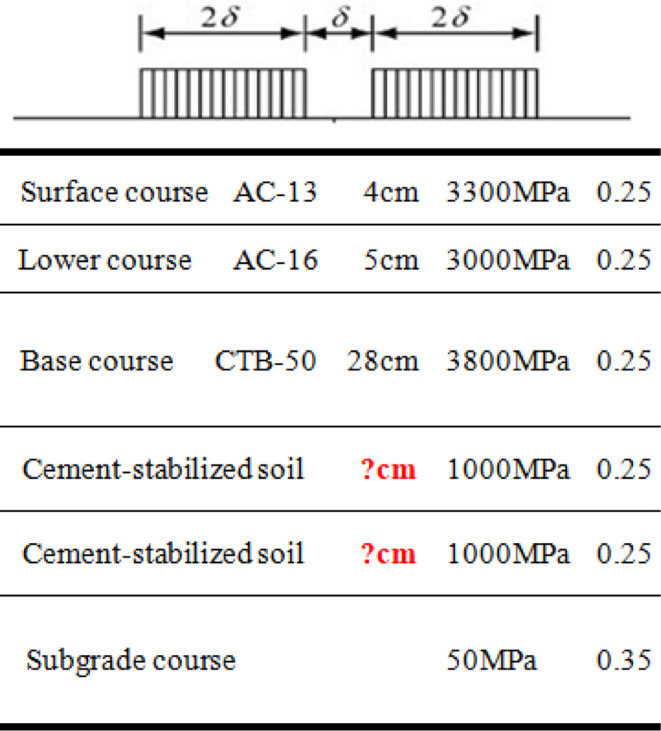
Mechanical calculation model of second–class highway pavement structure.

For this reason, it is recommended that the asphalt pavement surface course of second-class highways remain unchanged, CTB–50 base course of thickness 28 cm should be used, and a layer of construction should be executed. Based on this, the fatigue life of asphalt pavements with different cement-stabilized soil thicknesses was analyzed. The mechanical calculation model of the pavement structure is shown in Fig 15.

**Fig 16:**
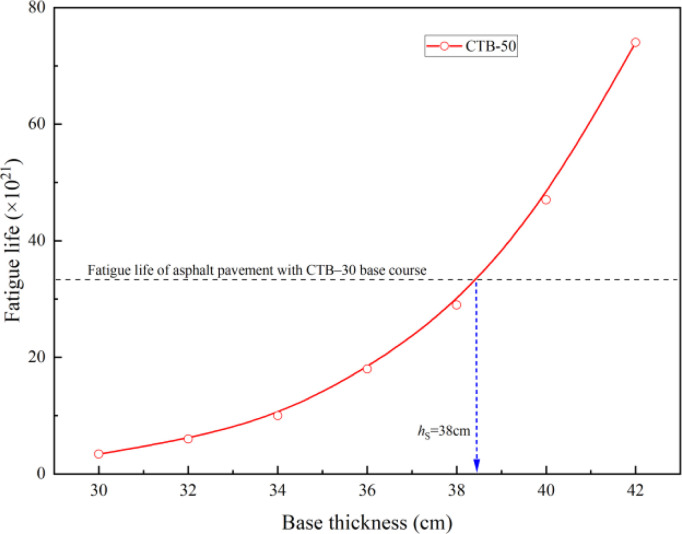
Variation of fatigue life with different cement-stabilized soil thicknesses of second highway.

The calculation results for the base layer bottom tensile stress of the asphalt pavement, stress level, and fatigue life of the asphalt pavement with different thicknesses of cement-stabilized soil are listed in Table 11. The fatigue life of the CTB–50 base layer varied with the thickness of the cement-stabilized soil, as shown in Fig 16。

**Table 11:** Mechanical calculation results of asphalt pavement with different thicknesses of cement-stabilized soil.

Items	Mechanical indicators of different cement-stabilized soil thicknesses (cm)						
	30	32	34	36	38	40	42
**Splitting strengths (MPa)**	1.57						
**Flexural strength (MPa)**	2.20						
**Base layer bottom tensile stress (MPa)**	0.2491	0.2424	0.2362	0.2304	0.2250	0.2200	0.2153
**Stress levels**	0.113	0.110	0.107	0.105	0.102	0.100	0.098
**Fatigue life (×10**^**21**^)	3.4	6	10	18	29	47	74

It can be observed from Fig 16 that if the second-class highway asphalt pavement is constructed with CTB–50 base course of thickness 28 cm, and the thickness of the cement-stabilized soil is increased from 30 to 38 cm, it can achieve the same fatigue life as the original pavement structure with a CTB–30 base course. The optimized second-class highway asphalt pavement structure with a CTB–50 base course is shown in Fig 16.

**Fig 17:**
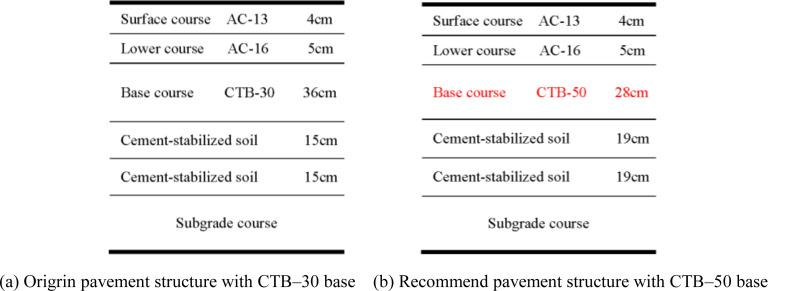
Recommended pavement structure of second–class highway. (a) Origrin pavement structure with CTB–30 base (b) Recommend pavement structure with CTB–50 base.

It can be noted from Fig 17 that the second-class highway asphalt pavement with a CTB–50 base course maintains the asphalt pavement layers unchanged; the base course of the asphalt pavement was transformed from a two-layer CTB–30 base course into a single-layer CTB–50 base course, and the thickness of each cement-stabilized soil course increased to 19 cm. This eliminates the construction of one layer of the base layer while ensuring the same thickness as a second-class highway asphalt pavement structure, thereby improving the overall integrity of the pavement structure.

## 6. Conclusion

The mechanical response and fatigue properties of asphalt pavements with CTB–50 and CTB–30 base courses were analyzed using BISAR 3.0 Subsequently, the typical pavement structure of asphalt pavement with a CTB–50 base course was recommended, and its effectiveness was verified through two highway engineering projects. The findings and conclusions are as follows.

(1) The BISAR software (version 3.0) was used to analyze the mechanical responses of different asphalt pavement structures. With constant pavement structure, the base layer bottom tensile stress of the asphalt pavement with CTB–50 base course increased by 6% compared to that of the asphalt pavement with CTB–30 base course. While the base structure decreases from a three-layer CTB–30 base course structure to a two-layer CTB–50 base course structure, the base layer bottom tensile stress of the asphalt pavement with a CTB–50 base course is reduced by 5% compared to that of the asphalt pavement with a CTB–30 base course.
(2) The influence of the base thickness on the tensile stress of the bottom layer of different asphalt pavement structures was analyzed. If the thickness of the single base layer increases, the base course of the asphalt pavement can be transformed from a three-layer CTB–30 base course into a two-layer CTB–50 base course, whereas if the thickness of the single base layer remains unchanged, the advantage of the CTB–50 base layer with large-thickness paving cannot be demonstrated, and the base layer bottom tensile stress increases.
(3) The fatigue properties of the asphalt pavements with CTB–50 and CTB–30 base courses were analyzed. The stress levels of the expressway and first-class highway asphalt pavement with a CTB–50 base course decreased by approximately 15% compared to that of the asphalt pavement with a CTB–30 base course; if the stress level is the same, the CTB–50 base layer thickness of asphalt pavement can be reduced by approximately 6 cm compared to that of the CTB–30 base layer.
(4) The typical pavement structure of an asphalt pavement with a CTB–50 base course was recommended based on two highway engineering projects. The recommended pavement structure can reduce the construction of one base layer and improve the integrity of the asphalt pavement structure. The experimental road paved with the CTB–50 base course showed no evident pavement disease, whereas the road section with the CTB–30 base course showed early crack disease in the asphalt pavement. In the future, more experimental roads with a CTB–50 base course will be paved, and their long-term performance will be tracked and observed.
